# CFTR bearing variant p.Phe312del exhibits function inconsistent with phenotype and negligible response to ivacaftor

**DOI:** 10.1172/jci.insight.148841

**Published:** 2022-03-22

**Authors:** Karen S. Raraigh, Kathleen C. Paul, Jennifer L. Goralski, Erin N. Worthington, Anna V. Faino, Stanley Sciortino, Yiting Wang, Melis A. Aksit, Hua Ling, Derek L. Osorio, Frankline M. Onchiri, Shivani U. Patel, Christian A. Merlo, Kristina Montemayor, Ronald L. Gibson, Natalie E. West, Amita Thakerar, Robert J. Bridges, David N. Sheppard, Neeraj Sharma, Garry R. Cutting

**Affiliations:** 1 Johns Hopkins University, Baltimore, Maryland, USA.; 2 University of North Carolina at Chapel Hill, Chapel Hill, North Carolina, USA.; 3 Seattle Children’s Research Institute, Seattle, Washington, USA.; 4 California Department of Public Health, Genetic Disease Screening Program, Richmond, California, USA.; 5 University of Bristol, Bristol, United Kingdom.; 6 University of Washington, Seattle, Washington, USA.; 7 Rosalind Franklin University of Medicine and Science, Center for Genetic Diseases, North Chicago, Illinois, USA.

**Keywords:** Genetics, Genetic variation

## Abstract

The chloride channel dysfunction caused by deleterious cystic fibrosis transmembrane conductance regulator (*CFTR*) variants generally correlates with severity of cystic fibrosis (CF). However, 3 adults bearing the common severe variant p.Phe508del (legacy: F508del) and a deletion variant in an ivacaftor binding region of CFTR (p.Phe312del; legacy: F312del) manifested only elevated sweat chloride concentration (sw[Cl^–^]; 87–105 mEq/L). A database review of 25 individuals with F312del and a CF-causing variant revealed elevated sw[Cl^–^] (75–123 mEq/L) and variable CF features. F312del occurs at a higher-than-expected frequency in the general population, confirming that individuals with F312del and a CF-causing variant do not consistently develop overt CF features. In primary nasal cells, CFTR bearing F312del and F508del generated substantial chloride transport (66.0% ± 4.5% of WT-CFTR) but did not respond to ivacaftor. Single-channel analysis demonstrated that F312del did not affect current flow through CFTR, minimally altered gating, and ablated the ivacaftor response. When expressed stably in CF bronchial epithelial (CFBE41o^–^) cells, F312del-CFTR demonstrated residual function (50.9% ± 3.3% WT-CFTR) and a subtle decrease in forskolin response compared with WT-CFTR. F312del provides an exception to the established correlation between CFTR chloride transport and CF phenotype and informs our molecular understanding of ivacaftor response.

## Introduction

Cystic fibrosis (CF) results from nucleotide alterations in the cystic fibrosis transmembrane conductance regulator (*CFTR*) gene, which encodes an ATP-binding cassette transporter that functions as an ATP-gated anion channel in epithelial tissues (1). Although over 2000 alterations have been reported in *CFTR* (http://www.genet.sickkids.on.ca/cftr/), variants that cause the loss of a single amino acid (in-frame deletions) play a disproportionate role in pathogenicity. Cystic fibrosis is primarily due to the prevalence of the in-frame deletion variant c.1521_1523delCTT (p.Phe508del; legacy: [delta]F508 or F508del) that is found in 70% of CF alleles and appears in 1 or 2 copies in approximately 85% of individuals with CF in the US (1). Other less common in-frame deletions in *CFTR* have also been characterized as pathogenic: c.1519_1521delATC (p.Ile507del; legacy: [delta]I507 or I507del); c.3011_3019delCTATAGCAG (p.Ala1004_Ala1006del; legacy: 3141del9 or 3143del9); and c.3067_3072delATAGTG (p.Ile1023_Val1024del; legacy: 3199del6) (https://cftr2.org) (2). Like F508del (3–5), these variants are associated with a classic presentation of CF, as the loss of an amino acid typically disrupts CFTR folding and transports to the apical surface of the epithelium, and significantly impairs its function. Consequently, the mild CF phenotype associated with the in-frame deletion c.935_937delTCT (p.Phe312del; legacy: [delta]F311 or F312del) in the CFTR2 database (https://cftr2.org) seemed incongruous. Although the F312del variant was originally reported in association with classic CF (6), it was later also identified in patients with milder disease (pancreatic sufficiency) (7, 8) and has not been mechanistically characterized. F312 lies in the fifth transmembrane (TM) segment of CFTR in a repetitive sequence of 3 phenylalanines at positions 310–312, just under 200 codons upstream of F508del.

We had compelling reasons to investigate F312del. First, detailed clinical evaluation of 3 individuals who harbored F312del and F508del revealed elevated sweat chloride concentration in the absence of any other features of CF. Second, 25 individuals in CF databases reported to have F312del as a presumed pathogenic variant had consistently elevated sweat chloride, yet highly variable lung and pancreas features. Third, the frequency of F312del in the healthy population (https://gnomad.broadinstitute.org/) is higher than predicted compared with the worldwide CF population in the CFTR2 database. Fourth, the 312 position is involved in the binding of 2 CFTR potentiators: ivacaftor and GLPG1837 (9–11). Thus, in-depth evaluation of the clinical and functional effects of F312del would likely inform understanding of the relationship between CFTR structure and function.

## Results

### Clinical evaluation of 3 individuals bearing F312del and F508del reveals elevated sweat chloride concentration in the absence of diagnostic features of CF.

**Figure 1:**
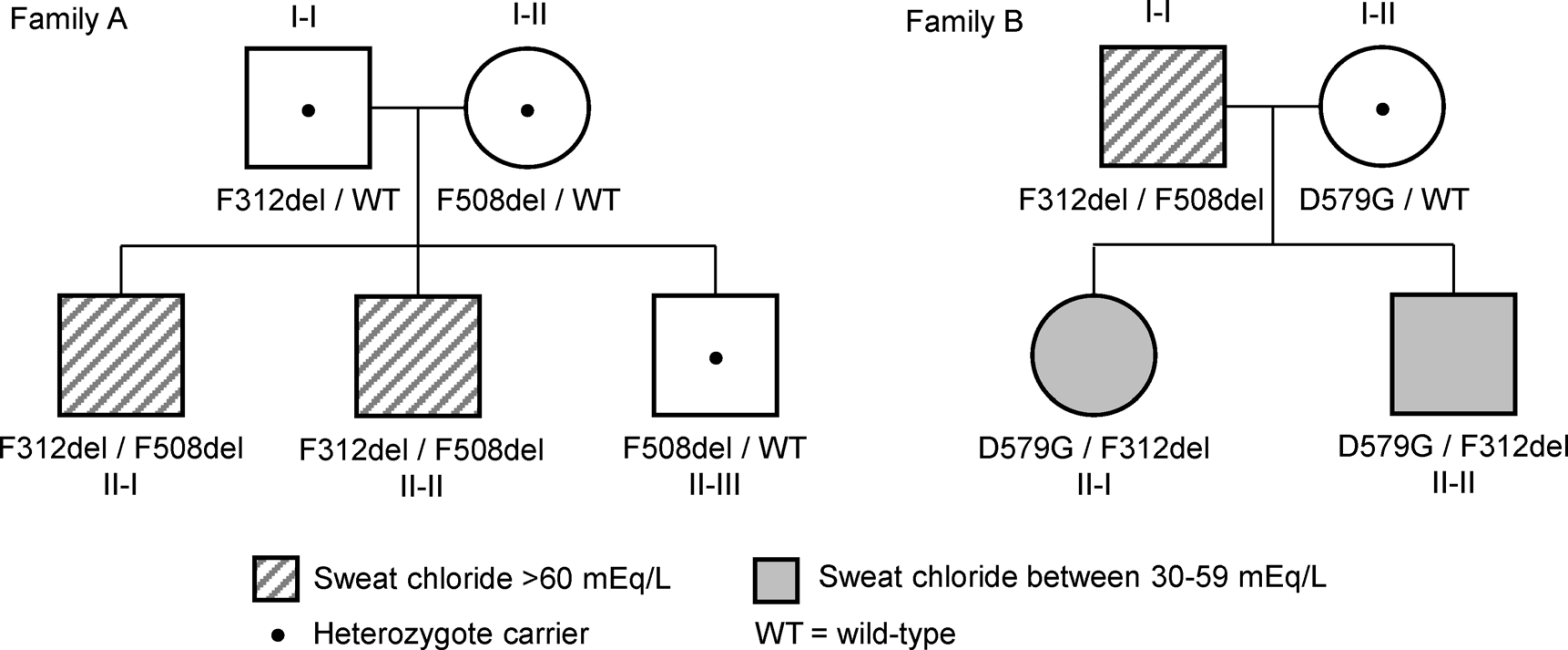
Pedigrees of 2 families segregating the F312del *CFTR* variant. Individuals II-I and II-II (F312del/F508del) from Family A were diagnosed following elevated sweat chloride testing, which was precipitated by the suspicion of CF-related liver disease in individual II-I. Individual I-I in Family B (F312del/F508del) underwent *CFTR* genotyping following a positive NBS for CF in his daughter. He was subsequently shown to have a diagnostic sweat chloride concentration. *CFTR* genotypes are shown below each family member.

We studied 2 families with individuals harboring F312del and F508del. In Family A, 2 males (II-I and II-II) inherited F312del from their father, and the father in Family B (I-I) transmitted F312del to his children (Family B: II-I and II-II) (Figure 1). The transmission pattern in these families confirmed that F312del and F508del were in different *CFTR* genes. Individual II-I (Family A) had hepatic cirrhosis and splenomegaly at 15 years of age, raising the possibility of CF-related liver disease. Sweat testing revealed elevated chloride concentrations (101/100 mEq/L). Subsequently, his brother (Family A: II-II) was found to have the same *CFTR* genotype and an elevated sweat chloride value. These siblings have forced expiratory volume in 1 second (FEV_1_) percent predicted of 75% and 77%; however, individual II-I’s spirometry is a restrictive pattern, thought to be due to chest wall deformities, while that of individual II-II is of an obstructive pattern attributed to mild asthma. Both siblings have normal lung parenchyma on chest CT (no structural abnormalities or bronchiectasis) and neither has classic manifestations of CF (e.g., sputum production, typical respiratory bacteria associated with CF, sinusitis, exocrine pancreatic insufficiency, or vas deferens anomalies as determined by testicular ultrasound; Table 1). Their diagnosis of CF based on elevated sweat chloride and *CFTR* genotype is now thought to be incorrect, particularly considering a recent genetic diagnosis from exome sequencing in individual II-I (Family A), which provides an alternative explanation for his liver disease (diagnosed on biopsy as cryptogenic cirrhosis).

The second family (Family B) was identified when individual II-I was diagnosed with CFTR-related metabolic syndrome (CRMS) based on elevated newborn immunoreactive trypsinogen (IRT), nondiagnostic sweat chloride values (< 60 mEq/L), and a *CFTR* genotype that includes variants not known to be definitively CF-causing (F312del and c.1736A>G [p.Asp579Gly; legacy: D579G]). Individual II-I and her brother (Family B: II-II) have normal to modestly elevated sweat chloride (range 25–51 mEq/L), consistent with the high residual function of CFTR bearing D579G (https://cftr2.org/). Parental testing revealed that her asymptomatic (and fertile) father (Family B: I-I) harbors F312del and F508del. Although sweat chloride concentration was elevated (87/90 mEq/L), the father has no evidence of lung disease nor other CF-related symptoms (Table 1). However, he had at least 3 episodes of heat exhaustion accompanied by nausea and vomiting during 6 months of intense physical training in the summer of 2012. Treatment with intravenous fluids was required on 2 occasions. Laboratory studies from the first episode revealed low serum sodium (127 mmol/L; reference 137–145), chloride (69 mmol/L; reference 98–107), and potassium (2.8 mmol/L; reference 3.6–4.8) and high glucose (127 mg/dl; reference 65–105) and creatinine phosphokinase (311 U/L; reference 30–135). During a subsequent episode, low sodium (135 mmol/L) and chloride (88 mmol/L) but normal glucose (82 mg/dL) were noted. A mild reduction in renal glomerular filtration rate was recorded on the first episode (54 mL/min/1.73m^2^) that was normal (> 60) during the subsequent episode. Thus, all 3 individuals harboring F312del and F508del manifest abnormalities in sweat chloride concentration without features of CF in the lungs, pancreas, or male reproductive structures.

### Individuals bearing F312del who are enrolled in CF databases consistently have elevated sweat chloride concentrations but other features of CF are highly variable.

Two subjects from the EPIC Observational Study (12) were found to carry F312del. Subject C (F312del and F508del) was evaluated at age 7 months due to failure to thrive and malnutrition. She had an elevated sweat chloride concentration and was treated with pancreatic enzymes. Her FEV_1_ at age 18 was 80% predicted (Table 1). Subject D was identified following an abnormal newborn screening (NBS) result and elevated sweat chloride concentration and was found to be homozygous for F312del and the intron 9 variant c.1210-7_1210-6delTT (legacy: 5T;TG11). Lung and pancreatic disease, *Pseudomonas aeruginosa* infection, and CF-related diabetes were absent at 12 years old (Table 1). Clinical findings in 17 individuals bearing F312del and a severe CF-causing variant enrolled in CFTR2 revealed that all had diagnostic sweat chloride concentrations (mean 91 mEq/L; range 75–123 mEq/L), with 56% using pancreatic enzymes and variable pulmonary function (mean FEV_1_ of 89% predicted; range 29%–127%). Finally, 6 individuals with F312del and a severe CF-causing variant identified by the California CF NBS program had elevated sweat chloride concentrations (mean 97 mEq/L; range 75–112 mEq/L; Table 1). A total of 5 of the 6 newborns had elevated IRT ranging from 80–223 ng/mL but only 1 is treated with pancreatic enzymes. The sixth newborn had a normal IRT (34 ng/mL) but was reported as a false negative after hospital admission for a respiratory virus with jaundice and cholestasis prompted clinical evaluation, which revealed elevated sweat chloride concentration and presence of F312del and F508del. Additional clinical details about the California CF NBS cohort are presented in Supplemental Table 1 (supplemental material available online with this article; https://doi.org/10.1172/jci.insight.148841DS1). In summary, individuals bearing F312del and either F508del or another bona fide CF-causing variant or F312del in homozygosity had consistently elevated sweat chloride concentrations but otherwise variable symptoms of CF.

### F312del occurs at a higher-than-expected frequency in the healthy population.

We compared the frequency of F312del in the healthy population to the affected (CF) population. Using the principle of Hardy-Weinberg equilibrium, we found that F312del occurs 22 times more frequently in the healthy population (expected frequency 3.41*10^–6^; actual frequency 7.57*10^–5^) (https://gnomad.broadinstitute.org/variant/7-117180210-CCTT-C?dataset=gnomad_r2_1) than estimated by its frequency in the CFTR2 population (details available in Supplemental Material). The gnomAD population, composed of individuals with “normal” phenotypes (13), also included 1 F312del-homozygous individual. The higher-than-expected frequency of F312del in the healthy population is consistent with the observation that only a fraction of individuals with F312del develops overt symptoms of CF.

### F312del is a recurrent variant existing on at least 2 genetic backgrounds.

While ascertaining the complete *CFTR* genotype associated with F312del, we noted that it occurred with 3 different versions of the polythymidine (polyT) and TG repeat tracts in intron 9 (14). Five individuals compound heterozygous for F312del and a CF-causing variant had *CFTR* intron 9 polyT or polyT;TG typing reported (Table 1). Under the assumption that F508del occurs exclusively with c.1210-13G>T (legacy: 9T;TG10) (15), it can be inferred that F312del occurs on 2 different backgrounds, including c.1210-13_1210-12dup (legacy: 7T;TG12) and c.1210-7_1210-6dup (legacy: 9T;TG11) (Table 1 and Supplemental Table 1). Furthermore, Patient D is homozygous for F312del and 5T;TG11, indicating that F312del occurs on a third genetic background. Whole genome sequencing performed on Subjects C and D (Table 1) allowed for in-depth analysis of the *CFTR* variants associated with F312del (i.e., haplotype). We were unable to assign which of Subject C’s 2 haplotypes associated with F312del but could unambiguously assign Subject D because he was homozygous for F312del, with identical *CFTR* haplotypes. Notably, the haplotype found in Subject D did not occur in Subject C, confirming that F312del occurs on different backgrounds, likely due to recurrent mutation (Supplemental Figure 1). The numerous single nucleotide variants that differed between the haplotypes provide evidence that F312del has occurred at least twice, probably due to its location within 3 adjacent and identical phenylalanine codons. Repeated sequence elements can lead to slipped strand mispairing, a mechanism known to expand or contract repeated DNA elements (16). The observation that F312del is recurrent is important, as the variant may exist in *CFTR* genes bearing other CF-causing variants.

### CFTR bearing F312del exhibits substantial function in human nasal epithelial cells.

**Figure 2:**
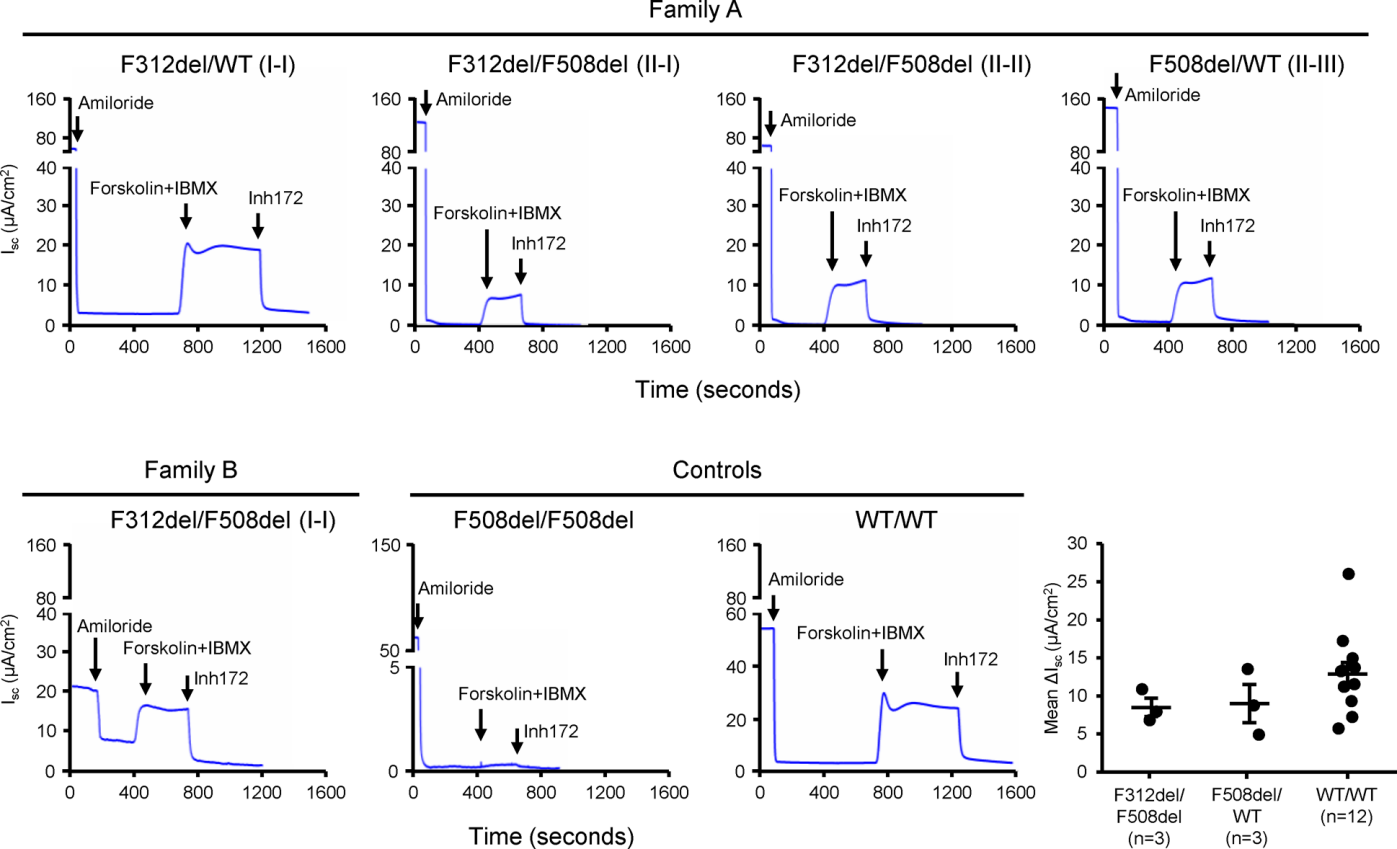
CFTR bearing F312del retains substantial function in well-differentiated ALI cultures derived from conditionally reprogrammed HNE cells. Representative I_sc_ recordings in HNE cells from individuals in Families A and B and from controls. *CFTR* genotypes are indicated. Amiloride (100 μM, apical) was used to inhibit the ENaC. CFTR function was measured as the difference (Δ) between current after stimulation with forskolin (10 μM, apical) and IBMX (100 μM, apical) to baseline following addition of the CFTR inhibitor CFTR_inh_-172 (Inh172; 10 μM, apical). The lower right panel shows ΔI_sc_ mean ± SEM from 4 technical replicates of HNE cells from 3 individuals with F312del/F508del, 3 individuals with F508del/F508del, and 12 individuals who are apparently healthy WT/WT. No individuals were taking CFTR modulators at the time of nasal cell collection.

To assess the function of F312del-CFTR, we studied human nasal epithelial (HNE) cells from 4 individuals from Family A and individual I-I from Family B (Figure 1) and previously-collected HNE cells from healthy (WT/WT; *n* = 12) individuals, 2 additional carriers of F508del (F508del/WT), and an individual homozygous for F508del (F508del/F508del). Cells were mounted in Ussing chambers and short-circuit current (I_sc_) measured to determine CFTR function in each individual. Following inhibition of the epithelial sodium channel by amiloride, CFTR currents were stimulated by forskolin and 3-isobutyl-1-methylxanthine (IBMX) and then inhibited by the CFTR inhibitor CFTR_inh_-172 (Figure 2). Although we studied only 1 individual homozygous for F508del, the level of CFTR function (ΔI_sc_=0.212 μA/cm^2^) observed in primary nasal cells is consistent with other studies of primary airway cells from F508del homozygotes (17, 18). As the 3 F312del/F508del HNE samples had significantly higher CFTR chloride transport than F508del/F508del HNE samples, we attributed the increased function to F312del-CFTR (Figure 2). Furthermore, the mean CFTR_inh_-172 inhibited currents (ΔI_sc_) of the 3 F312del/F508del individuals and the 3 F508del/WT individuals were similar, indicating that F312del-CFTR has substantial residual function (Figure 2). In addition, the CFTR current generated in primary nasal cells from the single F312del/WT individual (Family A: I-I) was in the range observed in the 12 WT/WT individuals, consistent with high residual function of F312del-CFTR. When normalized, CFTR function in the F312del/F508del individuals was 66.0% ± 4.5% of WT, which was attributed to F312del-CFTR as F508del-CFTR has less than 2% WT-CFTR function. Notably, the 12 WT/WT individuals did not have CFTR fully sequenced, so common variants with subtle functional effects may explain some of the variation within this group. Raw data underlying Figure 2 are available in the supplemental file Source Data.

### Acute treatment with ivacaftor does not increase CFTR function in primary HNE cells harboring the F312del variant.

**Figure 3:**
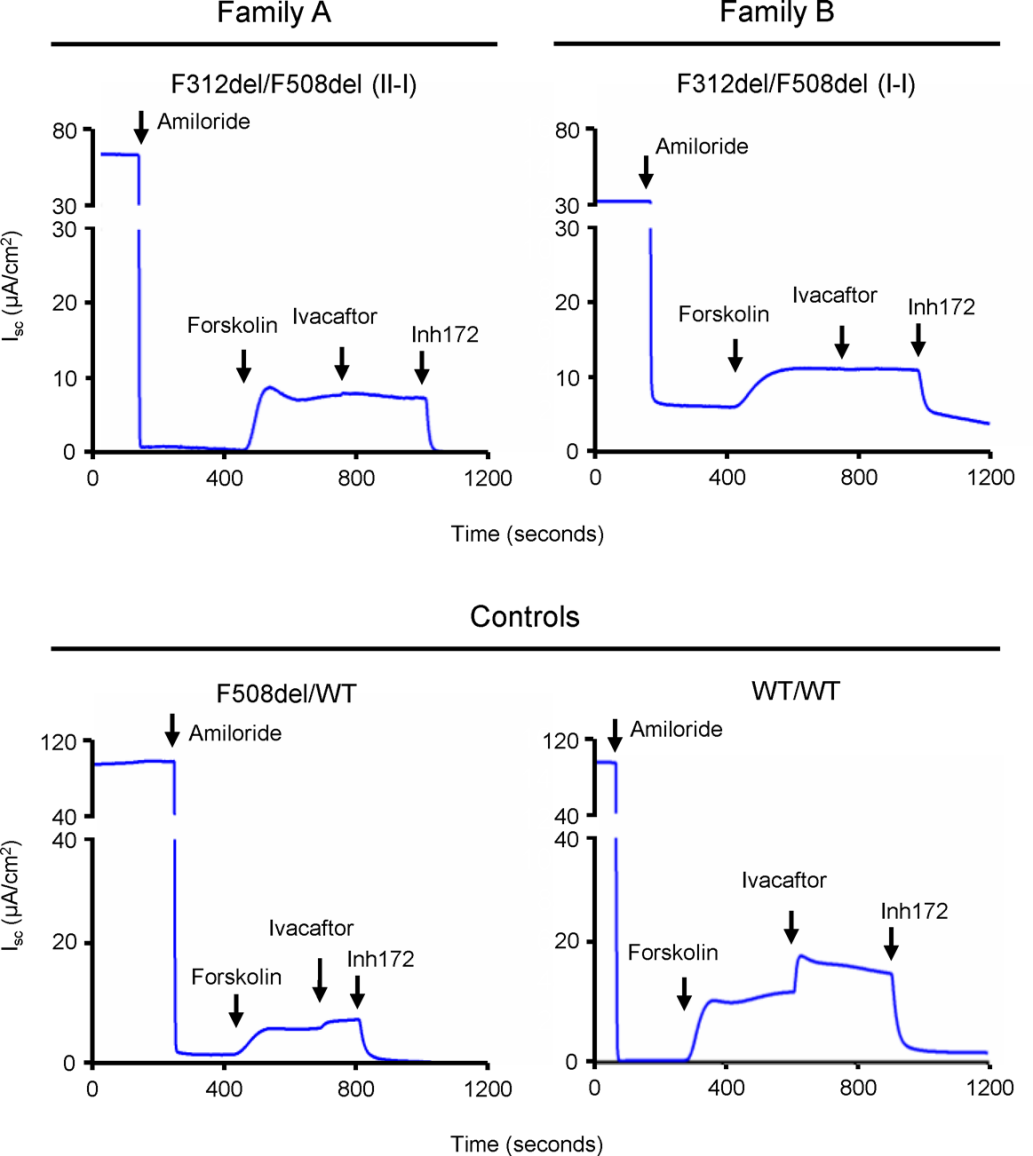
Ivacaftor does not potentiate function of CFTR bearing F312del in well-differentiated primary HNE cells. Representative I_sc_ recordings of HNE cells from individuals in Families A and B and from Controls. *CFTR* genotypes are indicated. Amiloride (100 μM, apical) was used to inhibit the ENaC. CFTR function was measured as the CFTR_Inh_-172 (Inh172; 10 μM, apical) inhibited current in HNE cells after stimulation with forskolin (10 μM, apical) and IBMX (100 μM, apical) and potentiation with ivacaftor (10 μM, apical).

Cryo-EM and biochemical studies have identified a binding site for the CFTR potentiator VX-770 (aka ivacaftor) in the CFTR protein formed by TM helices 4, 5, and 8 (9–11). Ivacaftor forms an aromatic interaction with the phenylalanine residue at codon 312 and substitution to alanine creates a synthetic CFTR mutant, p.Phe312Ala, that alters ivacaftor affinity and potentiator effect (9, 10). Therefore, we tested ivacaftor potentiation of CFTR function in primary HNE cells of individuals harboring F312del-CFTR. Acute ivacaftor treatment of HNE cells from 2 F312del/F508del individuals did not increase forskolin+IBMX–mediated CFTR function (–0.3 ± 0.5 μA/cm^2^). By contrast, ivacaftor application to HNE cells from WT/WT and F508del/WT individuals showed an increase in CFTR function of 2.3 ± 0.5 μA/cm^2^ and 2.5 ± 0.5 μA/cm^2^, respectively (Figure 3). The ivacaftor response in primary airway cells with 1 or 2 copies of WT-CFTR was significantly different from the mean response in F312del/F508del cells (*P* ≤ 0.001; 1-way ANOVA followed by Dunnett’s multiple comparison test). Since F508del-CFTR minimally responds to ivacaftor without prior treatment with CFTR correctors, the absence of effect on the F312del/F508del primary cells implies that F312del-CFTR also does not respond to ivacaftor.

### F312del-CFTR has minimally altered single-channel properties.

**Figure 4:**
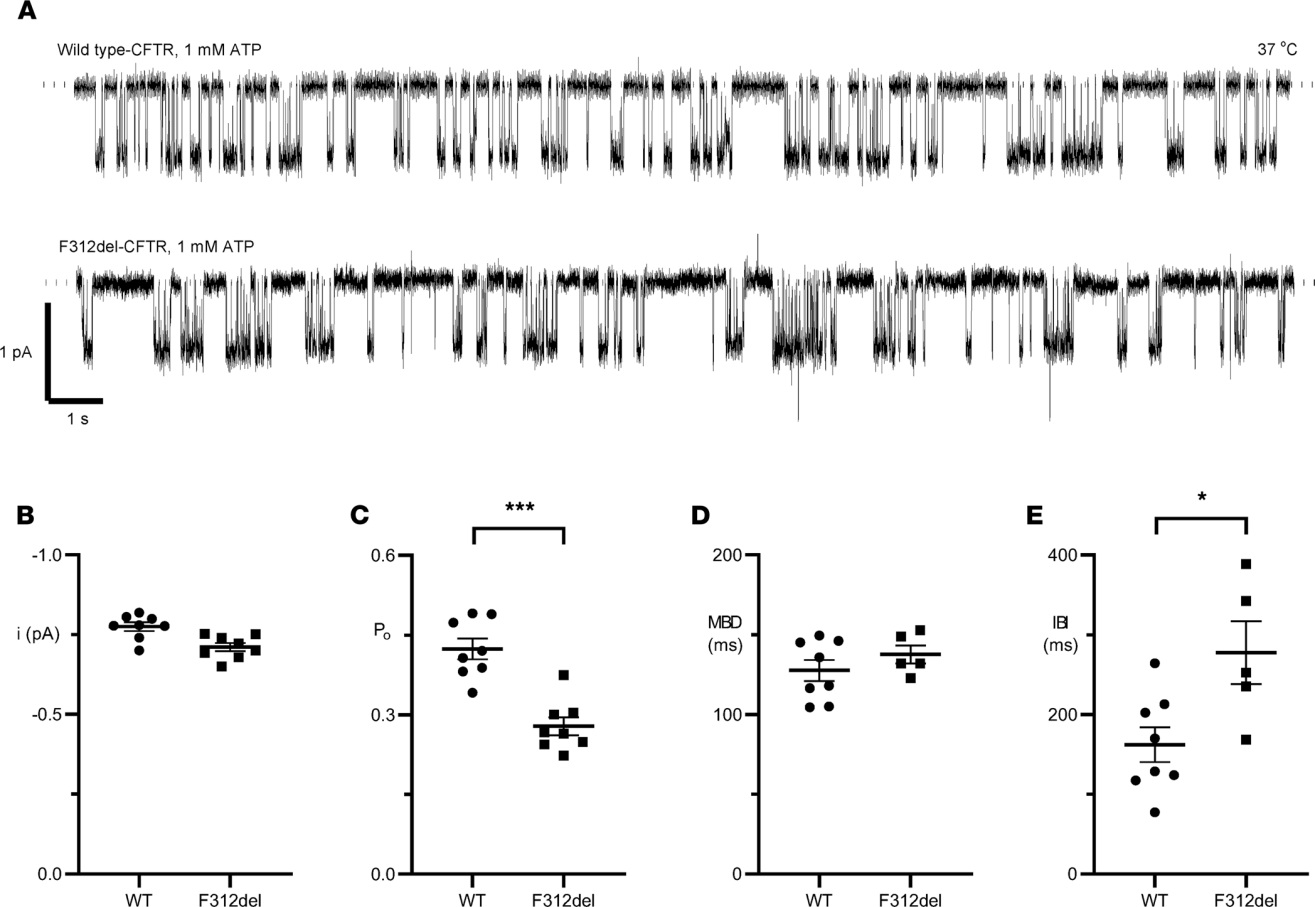
Single-channel behavior of WT- and F312del-CFTR. (**A**) Representative recordings of WT- and F312del-CFTR chloride channels in excised inside-out membrane patches. C127 cells stably expressing WT-CFTR and CHO cells transiently transfected with *CFTR* cDNA containing the F312del variant were used for the study. Cells were incubated at 37°C for 1–2 days prior to the study; for some experiments with F312del-CFTR, cells were then incubated at 27°C. The recordings were acquired at 37°C in the presence of ATP (1 mM) and PKA (75 nM) in the intracellular solution. Dotted lines indicate where channels are closed and downward deflections correspond to channel openings. A large chloride concentration gradient was imposed across membrane patches ([Cl^–^]_int_, 147 mM; [Cl^–^]_ext_, 10 mM) and membrane voltage was clamped at –50 mV. (**B**–**E**) Plots show *i*, *P*_o_, MBD, and IBI of WT- and F312del-CFTR. Data are means ± SEM (WT, *n* = 8; F312del, **B** and **C**, *n* = 8; and F312del, **D** and **E**, *n* = 5); ***P < 0.001 and *P < 0.05 versus WT-CFTR calculated using Student’s *t* test. For WT-CFTR, the number of active channels (N) per membrane patch = 1. For F312del-CFTR, *n* = 2–5 (*n* = 2 [2 membrane patches]; *n* = 3 [2 membrane patches], and *n* = 5 [4 membrane patches]). In 3 F312del-CFTR recordings, burst analysis was not possible because either too few bursts were acquired or the recordings were too noisy.

To further investigate F312del-CFTR function, we performed high-resolution single-channel recording in excised inside-out membrane patches. C127 cells stably expressing WT-CFTR and Chinese Hamster Ovary (CHO) cells transiently transfected with *CFTR* cDNA containing the F312del variant were used. Like WT-CFTR, F312del-CFTR exhibited bursts of channel openings interrupted by brief flickery closures but separated by longer closures between bursts (Figure 4A). We measured single-channel current amplitude (*i*), open probability (*P*_o_), mean burst duration (MBD), and interburst interval (IBI) (Figure 4, B–E). F312del was without effect on current flow through open channels, but modestly reduced *P*_o_ (36% reduction compared with WT-CFTR) by slowing channel opening without altering burst duration. These slight changes in single-channel behavior support our observations that F312del-CFTR retains significant apical membrane chloride channel function in airway epithelia. Raw data underlying Figure 4, B–E, are available in the supplemental file Source Data.

### Ivacaftor does not alter the single-channel properties of F312del-CFTR.

**Figure 5:**
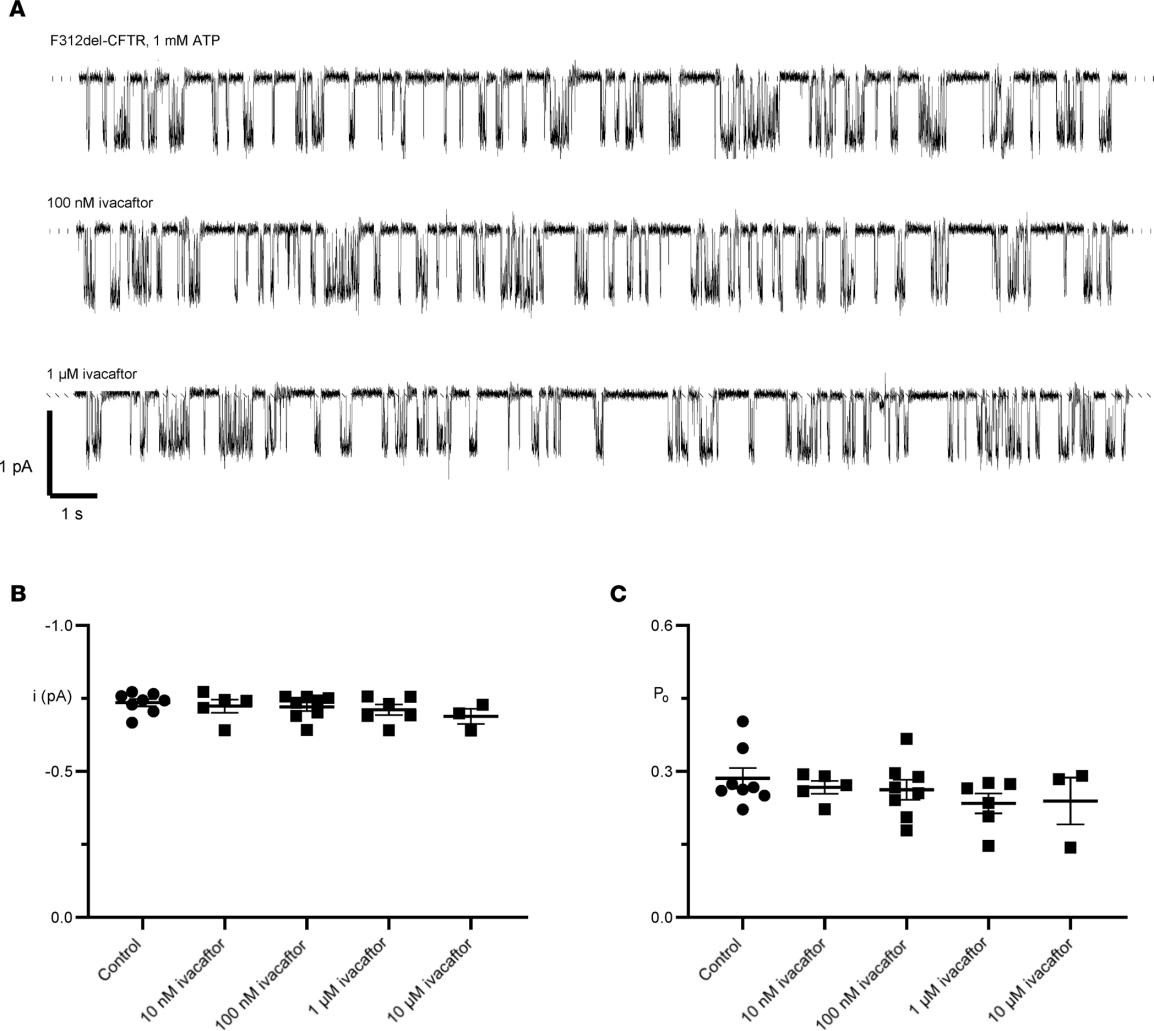
Single-channel behavior of F312del-CFTR in response to ivacaftor (VX-770). (**A**) Representative recordings of an individual F312del-CFTR Cl^–^ channel in an excised inside-out membrane patch from a transiently transfected CHO cell in the absence and presence of the indicated concentrations of ivacaftor (VX-770) in the intracellular solution. Dotted lines indicate where channels are closed and downward deflections correspond to channel openings. (**B** and **C**) Summary data show the effects of ivacaftor (10 nM–10 μM) on *i* and *P*_o_ of F312del-CFTR. Symbols represent individual values with mean ± SEM indicated (control, *n* = 8; 10 nM, *n* = 5; 100 nM, *n* = 8; 1 μM, *n* = 6; 10 μM, *n* = 3). There was no significant difference in the ivacaftor response calculated by 1-way ANOVA (**B**, *P* = 0.564; **C**, *P* = 0.494). Using similar experimental conditions, we previously demonstrated that ivacaftor (10 nM–10 μM) potentiates WT human CFTR (59).

To evaluate observations in primary HNE cells, we performed single-channel recordings of CHO cells expressing F312del-CFTR in the presence of increasing concentrations of ivacaftor (VX-770; Figure 5A). Neither the *i* nor *P*_o_ of F312del-CFTR were altered upon application of ivacaftor ranging from 10 nM to 10 μM (Figure 5, B and C). Of note, ivacaftor did not counter the reduced *P*_o_ of F312del-CFTR noted above. Since F312del-CFTR retains single channel properties similar to WT-CFTR, these results indicate that the F312del variant has a selective effect on ivacaftor interaction with CFTR, consistent with the lack of response seen in primary airway cells with F312del-CFTR. Raw data underlying Figure 5, B and C, are available in the supplemental file Source Data.

### F312del-CFTR is complex-glycosylated and retains substantial function in heterologous cells.

**Figure 6:**
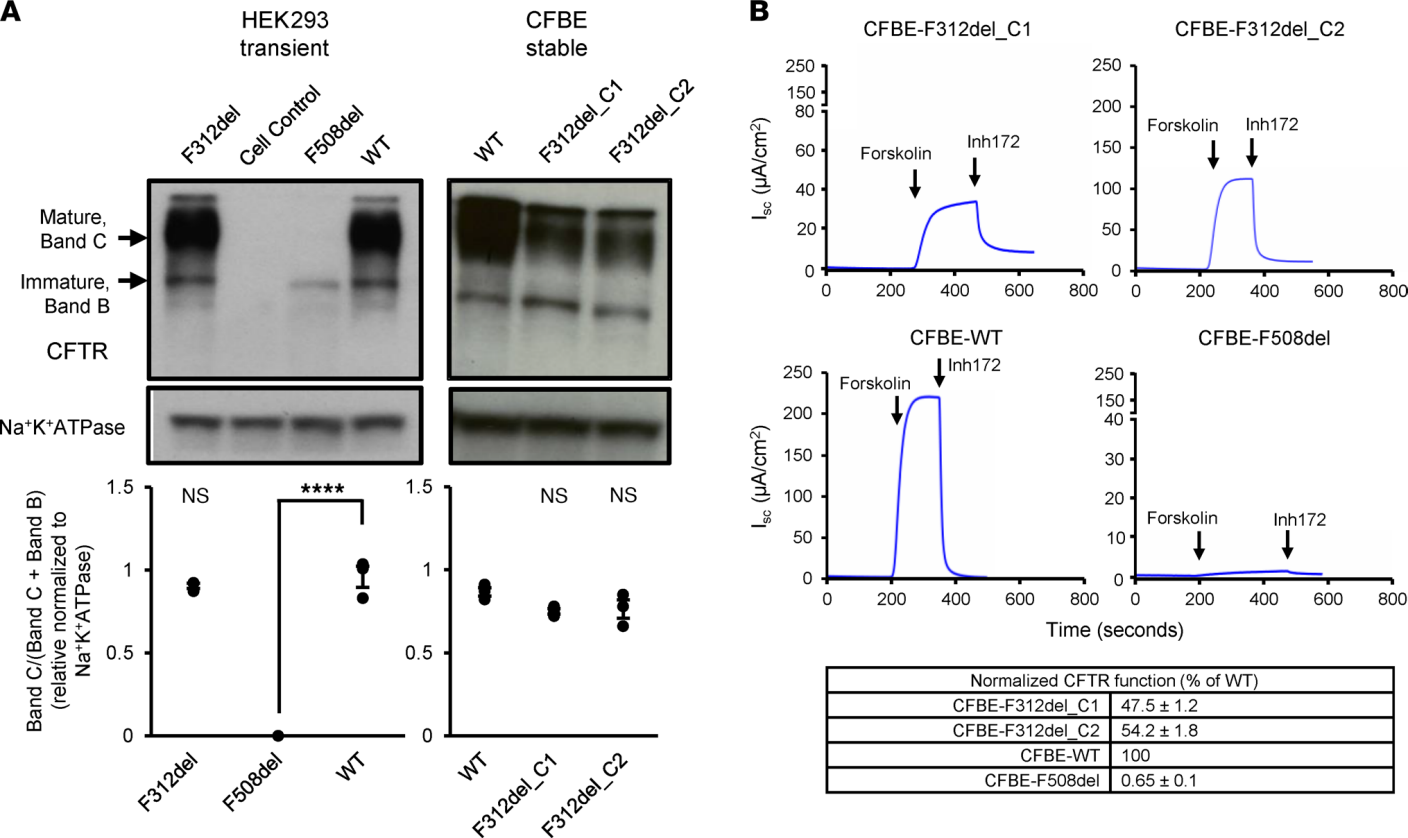
Protein processing and function of F312del-CFTR relative to WT-CFTR. (**A**) IB of protein lysates collected from HEK293 cells transiently transfected with F312del-CFTR, F508del-CFTR, WT-CFTR, and CAT-empty vector control. 40 μg of total cell lysates were electrophoresed and the IB probed with anti-CFTR antibody (596, Cystic Fibrosis Foundation Therapeutics). Anti-Na^+^K^+^ATPase (Abcam, ab76020) was used as loading control. Plots in lower panels show amount of mature CFTR protein relative to total CFTR protein normalized to the loading control using ImageJ (NIH) (*n* = 3 for each; 1-way ANOVA, ****P < 0.0001; ns, not significant). (**B**) Representative I_sc_ recordings in CFBE41o^–^ cells stably expressing either F312del-CFTR, WT-CFTR, or F508del-CFTR. We tested 2 different clones of F312del. After the baseline I_sc_ stabilized, forskolin (10 μM, basolateral) and CFTR_inh_-172 (Inh172; 10 μM, apical) were sequentially and cumulatively added at the indicated times. Individual I_sc_ recordings were acquired with Acquire and Analyze software (Physiologic Instruments) and plotted using GraphPad Prism 7.01 software. CFTR-specific function is defined as the difference (ΔI_sc_) between the sustained phase of the I_sc_ response after stimulation with forskolin and the baseline achieved after adding CFTR_inh_-172; this value is used to calculate CFTR specific function generated by a variant as described previously (19) and in the Supplemental Material.

To evaluate the effects of the F312del variant on CFTR processing, we performed IB analysis when F312del-CFTR was either transiently expressed in HEK293 cells or stably expressed in CF bronchial epithelial (CFBE41o^–^) cells. In both systems, WT-CFTR produced abundant mature, complex-glycosylated CFTR protein (band C) and some immature, core-glycosylated CFTR protein (band B) (Figure 6A). F312del-CFTR produced primarily mature complex-glycosylated protein, revealing no evidence of a folding defect and appearing markedly different than F508del-CFTR, which produced only band B (5) (Figure 6A). The estimated ratio of mature to immature CFTR generated by F312del-CFTR did not differ from WT-CFTR in either cell system (Figure 6A). To assess the function of F312del-CFTR relative to WT, we analyzed 2 clones of F312del-CFTR-expressing CFBE41o^–^ stable cell lines with the Ussing chamber technique and compared results with CFBE41o^–^ stable cell lines expressing WT-CFTR and F508del-CFTR (Figure 6B). When CFTR function was normalized for mRNA expression using correlations derived from 24 independent CFBE41o^–^ cell lines expressing WT-CFTR (see supplemental file Source Data and Raraigh et al. for details; ref. 19), F312del-CFTR clone 1 had 47.5% ± 1.2% and F312del-CFTR clone 2 had 54.2% ± 1.8% WT-CFTR function (mean ± SEM; 50.9% ± 3.3%), whereas F508del-CFTR generated less than 1.0% of WT-CFTR function. Thus, when heterologously expressed in mammalian cells, F312del-CFTR is efficiently folded to a fully-glycosylated mature protein and retains a considerable fraction of the cAMP-activated chloride transport observed for WT-CFTR. Raw data underlying Figure 6, A and B, are available in the supplemental file Source Data.

### F312del-CFTR displays a similar sensitivity to forskolin as WT-CFTR.

**Figure 7:**
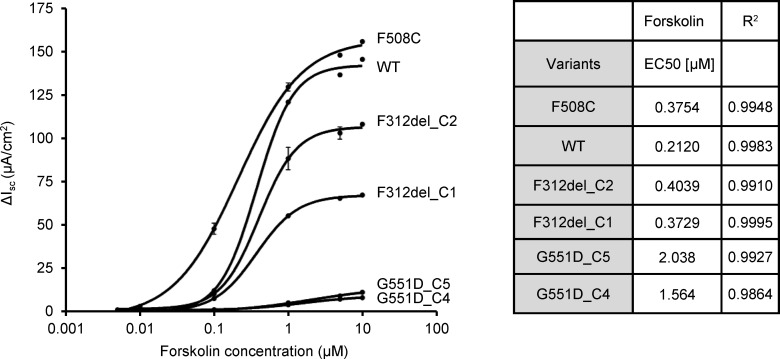
Forskolin dose response of CFTR variants expressed in CF airway cells. Chloride currents of CF airway cell lines stably expressing WT-CFTR or CFTR bearing variants F508C, F312del (2 clones: C1 and C2), or G551D (2 clones: C4 and C5) were measured by the I_sc_ assay. Forskolin concentration was increased from 0.005 μM to 10 μM and is plotted logarithmically. Data are means ± SEM (*n* = 3 for each). EC_50_ values were determined from I_sc_ measurements of each cell line using GraphPad Prism 9.2. R^2^ is the goodness of fit of the nonlinear regression.

Phosphorylation and dephosphorylation pathways differ between the sweat gland and other epithelial tissues (20). Given these organ-specific differences, we speculated that a substantially reduced response to forskolin might explain why individuals with F312del and a CF-causing variant manifest high sweat chloride concentration in the absence of severe lung or pancreatic disease. To assess this possibility, we determined the half-maximal effective (EC_50_) concentration of forskolin using dose response curves generated by sequential addition of forskolin to CFBE cells stably expressing F312del-CFTR (Figure 7). CFBE cells stably expressing G551D-CFTR, F508C-CFTR, and WT-CFTR were tested as controls. The F508C variant (c.1523T>G; p.Phe508Cys) does not cause CF when paired with a CF-causing *CFTR* variant and generates currents similar to WT-CFTR (https://cftr2.org), whereas the G551D variant (c.1652G>A; p.Gly551Asp) is a CF-causing variant with function of approximately 1% and known to respond well to ivacaftor. The EC_50_ of the 2 F312del clones were similar to each other (0.40 and 0.37 μM), despite clone 2 generating a higher current due to a higher level of expression (Figure 7). The EC_50_ of F312del was comparable to that of F508C-CFTR and both were slightly higher than WT-CFTR. By contrast, G551D-CFTR had a much higher EC_50_, as previously reported (21). Although we cannot exclude that F312del has a subtle effect on forskolin-mediated activation of CFTR, this difference is unlikely to explain the substantial elevation in sweat chloride concentration in individuals with F312del and a CF-causing variant (Table 1). Raw data underlying Figure 7 are available in the supplemental file Source Data.

## Discussion

Multiple studies have demonstrated that CFTR function below 25% WT-CFTR is associated with a CF phenotype that invariably includes elevation in sweat chloride concentration (22–24). Our data indicate that the F312del variant allows synthesis of mature, fully processed protein that generates chloride transport function estimated in the range of 66% ± 4.5% of WT-CFTR in primary HNE (mean ± SEM; *n* = 3 individuals) and 50.9% ± 3.3% WT-CFTR in immortalized CFBE stable cells (mean ± SEM; *n* = 2 clones). Varying levels of mRNA expression and lack of normalization or inclusion of IBMX could explain modestly higher estimates of F312del-CFTR function in primary cells, as the slightly lower level of WT-CFTR function determined in the CFBE stable cell system includes an adjustment for mRNA expression derived from 24 different WT clones (19). Based on the relatively preserved function, one would predict that an individual carrying F312del, even when paired with a CF-causing variant of very low function (e.g., F508del), would escape any manifestation of CF. Indeed, detailed clinical assessment by experienced CF physicians of 3 males carrying F312del and F508del revealed no evidence of CF-like disease in the lungs, pancreas, or vas deferens. However, all 3 had elevated sweat chloride concentrations. In addition, the father in Family B experienced multiple episodes of dehydration, volume depletion, and vomiting, very similar to the cases of heat prostration reported in children with CF during a summer heat wave in 1948 (25). As shown later, loss of salt from the sweat glands caused volume depletion and abnormally low serum sodium, chloride, and potassium in the children with CF (26), and we presume the same mechanism was responsible for the electrolyte abnormality episodes in the father of Family B. Of note are reports of 3 children who presented with dehydration and/or hypochloremic metabolic alkalosis (salt-loss syndrome) and were thereafter identified to have F312del (reported as ΔF311 or DF311) and other severe CF-causing variants (7, 8). Elevated sweat chloride concentration was a consistent feature in all 25 individuals with F312del paired with a CF-causing variant reported in databases. Thus, F312del presents a notable deviation from the robust correlation between CFTR chloride transport and sweat chloride concentration and the well-accepted correlation between elevated sweat chloride concentration and development of progressive CF disease leading to mortality (i.e., progressive obstructive lung disease and pancreatic exocrine insufficiency) (19, 27, 28).

The diagnosis of CF is based on the presence of characteristic clinical features along with evidence of CFTR dysfunction, which is typically demonstrated by an elevated sweat chloride concentration (29). A fraction of the 25 individuals in databases bearing F312del and a CF-causing variant warrant a diagnosis of CF as they exhibited exocrine pancreatic insufficiency and/or decreased pulmonary function (FEV_1_% predicted below 80%). Variable presence of lung and pancreatic disease may be due to environmental factors, such as exposure to second-hand smoke (30, 31) or inequitable receipt of healthcare related to socioeconomic status (32). Alternatively, other variants that affect CFTR function may be present in some but not all *CFTR* genes bearing F312del. Examples of in cis modifier variants have been reported in *CFTR,* the prime example of which is the c.350G>A (p.Arg117His; legacy: R117H) variant occurring with either the 7T or 5T variants in intron 9 (33). We observed that F312del occurs on a least 2 different genetic backgrounds and the presence of a third intron 9 T;TG variant in association with F312del (in the individual homozygous for F312del and 5T;TG11) could indicate a third unique background. In the case of Subject D, the 5T;TG11 variant might contribute to the pancreatic-sufficient CF phenotype observed by reducing efficiency of CFTR mRNA splicing, leading to reduced amounts of CFTR protein and lower rate of CFTR-mediated chloride transport. As we are not able to obtain T;TG tract status for individuals from the CFTR2 and California NBS cohorts, we cannot determine if this mechanism could account for their variable phenotypes. We did not identify other suspicious in cis variants following sequencing of the coding region of *CFTR* in the 3 probands from Families A and B or the entire *CFTR* gene in Subjects C and D. However, the apparent recurrence of F312del on multiple backgrounds, as has been previously postulated following identification of unique haplotypes on which F312del was found to reside (7), emphasizes that it may exist on other alleles containing *CFTR* variants beyond polyT variation; therefore, familial testing to determine the phase of the 2 identified *CFTR* variants is always recommended as part of a genetics evaluation. It is also possible that genetic variants independent of *CFTR* operate in some individuals but not others. Such genetic modifiers could affect cellular function so that approximately 50% WT-CFTR chloride transport is insufficient to avoid disease in airway or pancreatic epithelia, as may be the case for “symptomatic CF carriers” (34, 35). In striking contrast, individuals from nonscreened populations bearing F312del and a CF-causing variant with *only* elevated sweat chloride would not meet a diagnosis of CF, regardless of the presence of a CF-causing variant and F312del (classified as associated with varying clinical consequences; https://cftr2.org), because a diagnosis of CF requires clinical features of the disease. Whether heat prostration and/or hypochloremic dehydration associated with F312del represent a unique form of a CFTR-related disorder (CFTR-RD) specific to the sweat gland is unclear; these clinical findings are not currently accepted as CFTR-RDs nor do they rise to the level of clinical severity to diagnose CF. With the variability in clinical presentation, however, a careful comprehensive review of potential organ system involvement including and beyond what is typically done for diagnostic purposes needs to be undertaken to ensure that these individuals do not have manifestations of CF (spirometry or lung clearance index testing, chest CT or other imaging, pancreatic elastase assessment, testicular ultrasound, and additional testing as clinically indicated; functional testing using primary cells may also be considered). Given our results, we predict that elevated sweat chloride concentration is present in all or almost all individuals who carry F312del and a CF-causing variant, but many of these individuals are unlikely to be identified unless they have family members suspected of having CF, they experience episodes of “heat prostration,” or they are identified following CF NBS due to elevated IRT, which is known to occur even in heterozygous healthy carriers of pancreatic insufficient-CF–causing (PI-CF–causing) variants such as F508del. Indeed, our genetic analysis of the healthy population demonstrating an overabundance of F312del carriers (13) confirms that most individuals bearing F312del and a CF-causing variant do not have a diagnosis of CF.

How does a modest reduction in chloride transport elevate sweat chloride concentration? Our single-channel studies support the observation that F312del minimally affects CFTR chloride channel function; it was without effect on current flow but slowed channel opening to reduce *P*_o_ by about one-third, consistent with Ussing chamber studies of CFTR-mediated chloride transport. These studies suggest a Class III (defective regulation) (36) variant. However, the gating defect of F312del-CFTR is miniscule when compared with the archetypal Class III variant G551D-CFTR, characterized by dramatic prolongation of the closed time between channel openings and hence, profound attenuation of *P*_o_ (37). Elevated sweat chloride concentration despite considerable residual chloride channel function has been reported with variants that truncate the C-terminus of CFTR, such as c.4364C>G (p.Ser1455X; legacy: S1455X) and c.4426C>T (p.Gln1476X; legacy: Q1476X) (38–40). Notably, these C-terminal variants permit the synthesis and processing of truncated mature CFTR protein, which generates near WT levels of chloride transport (38, 41). Loss of the C-terminal PDZ-binding motif destabilizes CFTR at the plasma membrane, but whether altered tethering leads to elevated sweat chloride concentration is unknown (41, 42). Alternatively, F312del may affect G protein-induced activation of CFTR in sweat glands. Reddy and Quinton (43) have proposed that G proteins activate CFTR *G*_Cl_ in the native sweat duct, and it is unknown whether G protein-mediated activation of CFTR is applicable to all transporting cells (i.e., secretory as well as absorptive cells of the airways and sweat gland cells) or whether it is unique to cells performing an absorptive function in sweat duct cells. While beyond the scope of this manuscript, studies of the sweat glands may provide insight into to the mechanism of action of F312del upon CFTR function in this tissue.

Other properties of CFTR might be affected by F312del, such as mechanisms that facilitate bicarbonate transport (44–46). Abrogation of bicarbonate transport is an attractive explanation, as loss of carbonic anhydrase function, either by genetic mutation (e.g., *CA12*; ref. 47) or by pharmacologic inhibition (e.g., topiramate; ref. 48), is associated with increased chloride concentration in sweat. However, preliminary studies by our group suggest that F312del-CFTR transports bicarbonate at reduced rates that are proportional to the reduction in chloride transport. Alternatively, F312del may alter the activation of CFTR, leading to erroneously high estimates of function. However, the forskolin EC_50_ for F312del-CFTR was not substantially different from a non–CF-causing variant and only modestly higher than WT-CFTR. Other possibilities that have not been explored include altered interaction between CFTR and other proteins that transport ions. Of note, the missense variant c.2252G>T (p.Arg751Leu; legacy: R751L), located in the R domain of CFTR and associated with elevated sweat chloride and mild CF features in 3 related individuals was shown to have minimal effect on CFTR function. Interestingly, function of the epithelial sodium channel (ENaC) was reduced, suggesting that R751L may affect the interaction between CFTR and ENaC in the sweat gland (49). The 3 individuals with genotype F312del/F508del have different responses to amiloride, ranging 20–120 μA/cm^2^. Thus, further studies are needed to determine if F312del-CFTR has an altered interaction with ENaC.

The location of F312del within a region reported to interact with ivacaftor afforded another interesting aspect to this variant (9–11). F312 is 1 of 3 contiguous phenylalanine (Phe) residues (codons 310–312) in TM helix 4, the only such instance in CFTR. The Phe residue at codon 312 interacts with ivacaftor (7) and substitution to Alanine (p.Phe312Ala) alters ivacaftor binding and potentiation of CFTR (9–11). Lack of response of F312del-CFTR to ivacaftor supports the contention that the TM domain 1 (TMD1) region is the primary site of ivacaftor binding. In possible contrast to our results, F312del has been approved by the FDA for treatment with ivacaftor based on testing CFTR expressed heterologously in Fischer rat thyroid cells. It is possible that an ivacaftor response was observed due to high levels of CFTR expression that can be achieved in the heterologous cell system. However, the parameters and results of testing are not available. The response of primary cells from 2 unrelated individuals and the dose escalation in single-channel studies indicate that ivacaftor does not potentiate F312del-CFTR in vivo. Of note, the lack of detectable response to ivacaftor indicates that the remaining Phe residues are unable to coordinate with the oxyquinlone moiety of ivacaftor despite F312del-CFTR being processed to mature form and retaining considerable function.

Could a distinct structural feature, such as the TMD1 region that interacts with ivacaftor, have other functions essential for CFTR-mediated ion transport in the sweat gland? The ivacaftor binding region coincides with a hinge region in TM segment 8 that is unique to CFTR and appears to contribute to CFTR channel gating (9, 50). Since ivacaftor and the potentiator GLPG1837 can bind to the TMD1 region, is it possible that cellular proteins interact with CFTR via the same region to facilitate its localization in apical and basolateral membranes in the sweat duct? Could disruption of this motif by F312del alter an interaction essential for CFTR-mediated chloride reabsorption leading to elevated sweat chloride? Further work is needed to fully investigate these and other questions, but the informative exception to established correlations between *CFTR* genotype and CF phenotype and insight into potentiator binding provided by F312del illustrate the unique opportunities afforded by detailed study of select naturally occurring *CFTR* variants.

## Methods

Supplemental Methods are available online with this article.

### Clinical data collection.

We recruited 3 individuals compound heterozygous for F312del and F508del (2 siblings and an unrelated singleton) for phenotype and functional study; their care providers contributed clinical details. Clinical and demographic data from additional individuals bearing F312del in homozygosity or in presumed compound heterozygosity with a PI-CF–causing variant were collected from the Clinical and Functional TRanslation of CFTR (CFTR2) project (*n* = 17), the Cystic Fibrosis Genome Project (CFGP; *n* = 2), and the Genetic Disease Screening Program in California (*n* = 6). The CFTR2 project collected worldwide data from individuals with CF (51). The CFGP provided whole genome sequence (WGS) data and clinical details from 2 individuals bearing F312del and F508del or F312del in homozygosity who were originally recruited via the Early Pseudomonas Infection Control (EPIC) Observation Study (12). The Genetic Disease Screening Program in California (52) provided clinical details from 6 individuals who underwent CF NBS, were found to harbor F312del and a severe CF-causing variant, and were subsequently diagnosed with CF.

### Haplotype analysis.

Haplotype analysis was performed on WGS data to determine the molecular background of F312del in 2 individuals from the CFGP. Variants within *CFTR* with greater than 15% minor allele frequency in the study were phased on the University of Michigan Imputation Server with Eagle v2.4, using 1000G Phase3 v5 as the reference panel. The haplotypes were imputed using VCFtools v0.1.13.

### Assessment of F312del-CFTR expression and function.

Primary nasal cell culture, transient transfection, creation of stable cells, measurement of CFTR function, IB, and patch-clamp techniques have been described previously (19, 37, 53–58). Briefly, CFTR function was assessed by I_sc_ measurement in primary HNE cells, which were differentiated into polarized epithelia at an air-liquid interface (ALI). Conditionally reprogrammed primary cultures of HNE cells (passage 2) seeded onto permeable filter supports were maintained at the ALI for 21–28 days. The filter inserts were placed in Ussing chambers at the time of measurement and bathed on both sides with Krebs-Ringer bicarbonate solution bubbled with 95% O_2_/5% CO_2_ and maintained at 37°C. After the baseline I_sc_ stabilized, amiloride (100 μM, apical), forskolin/IBMX (10 μM and 100 μM, basolateral), and CFTR_inh_-172 (inh-172; 10 μM, apical) were sequentially and cumulatively added at the indicated times. I_sc_ recordings were acquired with Acquire and Analyze software (Physiologic Instruments) and plotted using GraphPad Prism 7.01 software. CFTR protein processing and function were tested in HEK293 and CFBE41o^–^ cells heterologously expressing F312del-CFTR. To investigate the single-channel behavior of F312del-CFTR, excised inside-out single membrane patches from transiently transfected CHO cells were used.

### Statistics.

Descriptive statistics (mean, standard error of the mean) were used to describe results from experimental measures of CFTR function or response to ivacaftor. Measured means were compared using either 2-tailed Student’s *t* test or 1-way ANOVA with the threshold of significance set at *P* > 0.05. Nonlinear regression goodness of fit (R^2^) was used for dose-response curves.

### Study approval.

This study was approved by the Institutional Review Boards at Johns Hopkins Medicine, Baltimore, MD (IRB00116966 and NA_00018599) and Seattle Children’s Hospital, Seattle, WA (IRB11686). We obtained written informed consent from all individuals, except for the California de-identified patient information, which is covered under a State of California, Health and Human Services Agency’s Committee for the Protection of Human Subjects exemption (CPHS15-02-1898).

## Author contributions

KSR conceived the overarching project goals, collected and contributed data, performed data analysis, and wrote the paper. KCP performed experiments, analyzed the data, and wrote the paper. SS collected and contributed data and reviewed and edited the paper. YW performed experiments and prepared and presented the data for the paper. MAA contributed data, performed data analysis, and prepared and presented data for the paper. HL contributed data and performed data analysis. DLO performed experiments and analyzed the data. AVF and FMO contributed data. JLG, ENW, SUP, CAM, KM, and AT collected and/or contributed data, including patient materials. RLG contributed data, reviewed and edited the paper, and provided oversight to other authors. NEW collected and contributed data, including patient materials, and reviewed and edited the paper. RJB and DNS oversaw the design of experimental methodology, verified experimental results, reviewed and edited the paper, provided oversight to other authors, and received funding that was used to complete experiments for this project. NS conceived the overarching project goals, collected and contributed data, performed data analysis, wrote the paper, prepared and presented data for publication, and provided oversight to other authors. GRC conceived the overarching project goals, wrote the paper, provided oversight to other authors, and received funding that was used to complete experiments for this project.

## Supplementary Material

Supplemental data

Supplemental data set 1

## Figures and Tables

**Table 1:**
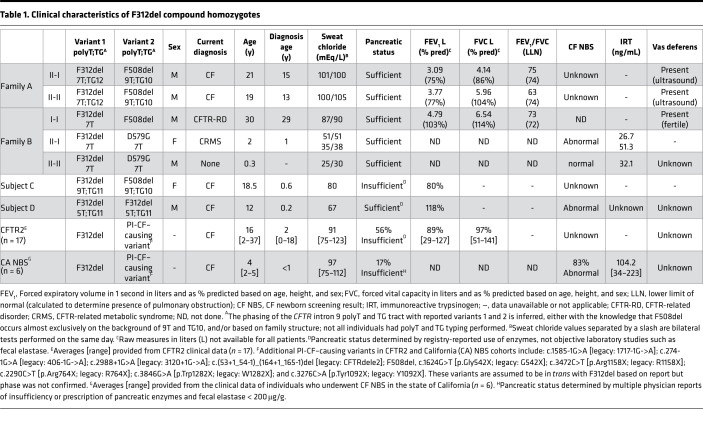
Clinical characteristics of F312del compound homozygotes

## Version 1 03/22/2022

Electronic publication
