# Supplementary material for: CFTR bearing variant p.Phe312del exhibits function inconsistent with phenotype and negligible response to ivacaftor
Source: JCI Insight. 2022 Mar 22;7(6):e148841. doi: 10.1172/jci.insight.148841 (PMC8986068; doi:10.1172/jci.insight.148841)
Supplement: Supplemental data [file jciinsight-7-148841-s135.pdf]

CFTR bearing variant p.Phe312del exhibits function inconsistent with phenotype and negligible response to ivacaftor

SUPPLEMENTAL MATERIAL: METHODS

Calculation of expected vs. actual frequency of F312del based on Hardy-Weinberg equilibrium

This calculation was based on the following equations:

$$p + q + x = 1$$

$$p^2 + 2pq + 2px + 2qx + q^2 + x^2 = 1$$

In which:

- p (wild-type frequency) = 0.982461356
- q (other CFTR variant frequency, not including F312del) = 0.017535170
- x (F312del frequency) = 0.00000347

The frequency of F312del (x) was derived from its allele count in the CFTR2 dataset (28 of 141,340 alleles). Carrier frequency for CF in the general population was assumed to be 1 in 29 (1). The expected frequency of F312del carriers (2px) in the general population allowed estimation of the expected allele frequency.

Collection of primary nasal cells

Nasal cells were collected from CF-affected and healthy individuals following IRB protocol at Johns Hopkins University, Baltimore, Maryland (IRB# 00116966). An experienced physician performed endoscopic procedures to harvest nasal cells from the mid-part of the inferior turbinate of healthy and CF-affected individuals by brushing with interdental brushes after spraying a topical anesthetic on the nasal mucosa.

Isolation, expansion, and culture of primary human nasal epithelial cells

Expansion and culture of human nasal epithelial (HNE) cells were performed, as previously described (2-4). Briefly, HNE cells were expanded by culturing in propagation media (DMEM/F-12 -3:1) in the presence of 10 μM Y-27632 (Sigma #Y0503), a ROCK inhibitor, and irradiated

fibroblast feeder cells (Kerafast #EF3003). After 2 passages of expansion, cells were seeded (~3-5x10^5 cells/cm^2) onto Snapwell inserts (Costar #3801). On confluence (day 5-7), propagation media was replaced with differentiation media (DMEM/F-12 -1:1) containing Ultroser G serum substitute (Pall; Port Washington, NY) without reagent Y. It is noted that in one HNE sample, PneumaCult-ALI Basal Medium was used as differentiation media (Stem Cell Technologies #05002). The following day, cells were maintained at an air-liquid interface (ALI) by removing media from the apical compartment and providing media to the basal compartment only. The apical surface was washed with phosphate-buffered saline (PBS) to remove any mucus accumulation, and the medium was replaced in the basal compartment every 48 hours. Cells were maintained at 37°C and 5% CO_2. *CFTR* genotypes of each culture were confirmed by CFTR-specific PCR followed by Sanger sequencing of genomic DNA isolated from respective HNE cells.

Transient transfection

Human Embryonic Kidney (HEK293) cells were grown to ~95% confluency on six-well plates containing growth medium (DMEM [ThermoFisher Scientific #11965118]) supplemented with 10% fetal bovine serum (Corning # 35-011-CV) and 1% penicillin-streptomycin (ThermoFisher Scientific #15140122) and kept at 37 ^oC in a 5% CO_2 incubator. The cells were transfected with 4 µg of pcDNA5FRT plasmid harboring either wild-type (WT) or mutant CFTR and 8 µl of Lipofectamine2000 in Opti-MEM according to the manufacturer's instructions (ThermoFisher Scientific #11668027). Non-transfected parental cells were used as a negative control. Cells were prepared for immunoblot analysis at 48 hours post-transfection.

Immunoblot analysis

Total protein was isolated from cells using cOmplete Lysis-N solution (Roche #04719956001) containing protease inhibitor cocktail tablets (Roche #16858101). The lysates were centrifuged and protein concentrations were estimated using the Microplate BCA Protein Assay kit (Thermo Scientific #OF184596) according to the manufacturer's instructions. The lysates were subjected to immunoblot analysis. Briefly, protein samples were subjected to immunoblotting by denaturing 40 µg of protein at 37 °C for 30 min in 4X Laemmli sample buffer (Biorad) containing

200 mM DTT. Samples were resolved on 7.5% Mini-PROTEAN® TGX™ precast gels (BioRad, Hercules, CA, USA) and transferred to a PVDF membrane (Biorad #BR20160914) membrane using a Bio-Rad Trans-blot turbo semidry transfer system (Biorad) set at 2.5 mA/25 V/10 minutes. Membranes were blocked in 5% nonfat dry milk, Phosphate buffered saline (PBS), and 1% Tween-20 (PBS-T) at room temperature for 1 hour before being exposed to an antibody overnight at 4 °C that recognized human CFTR (CFFT #596, 1:5000) (5). Equivalent protein loading between the samples was verified by probing membranes for Na^+K^+ATPase (abcam #EP1845Y, 1:50000) overnight at 4 °C. Membranes were exposed to anti-mouse and anti-rabbit secondary antibodies conjugated with horseradish peroxidase at a dilution of 1:150,000 in PBS-T for 1 hour at room temperature to detect CFTR and Na^+K^+ATPase, respectively. Signals were detected with ECL Prime chemiluminescence detection system (GE Healthcare, Pittsburgh, PA, USA), and exposure to x-ray film (GE Healthcare #28-9068-39).

Generation of CFBE stable cell lines

CF bronchial epithelial (CFBE41o$^-$) containing a Flp Recombinase Target (FRT) integration site, which facilitates site-specific recombination, were used to create stable cell lines expressing F312del-CFTR as described previously (6, 7). Wild type-*CFTR* pcDNA5/FRT plasmid (as previously described) (8) was used as a template for site-directed mutagenesis for F312del-CFTR. The entire *CFTR* cDNA was confirmed to not contain secondary mutations by Sanger Sequencing. CFBE cells were co-transfected with *pOG44* recombinase plasmid (ThermoFisher Scientific #V600520) and variant plasmid, then grown under 200 mg/ml hygromycin selection from individual clones isolated with cloning cylinders (Sigma #C1059). Integration of the entire CFTR cDNA and the flip-in site was confirmed by PCR amplification of genomic DNA from clonal cell lines. Expression of CFTR in each cell line was evaluated by qRT-PCR, as described previously (7).

Assessment of CFTR function by short-circuit current measurement

CFBE stable cells

To evaluate the functional consequence of CFTR-specific chloride conductance, cells (2.5-4x10^5) were plated onto Snapwell filters (Corning Costar #3407; 12 mm filter diameter with 0.4 µm pore diameter) and were fed daily for six days from both basolateral and apical sides with DMEM (High glucose; GibcoTM #11965092) with 10% fetal bovine serum (Corning # 35-011-CV) and 1% penicillin/streptomycin (ThermoFisher Scientific #15140122). Using an Epithelial Voltohmmeter (EVOM, World Precision Instrument), transepithelial resistance (R_t) was measured every day using Epithelial Volthohmmeter (EVOM, World Precision Instrument). After approximately six days, a R_t of ~ 180-200 Ω was achieved. When epithelia achieved a R_t >200 Ω, filters were mounted into EasyMount Ussing chambers to measure the short circuit current (I_{sc}) with a VCC MC6 or VCC MC8 multichannel voltage-current clamp amplifier (Physiologic Instruments). To quantify CFTR-mediated I_{sc}, CFBE epithelia were bathed in nonsymmetric buffers without permeabilizing the basolateral membrane. The basolateral buffer was composed of 145 mM NaCl, 1.2 mM $MgCl_2$, 1.2 mM $CaCl_2$, 10 mM dextrose & 10 mM HEPES (pH = 7.4) and the apical buffer was composed of 145 mM NaGluconate, 1.2 mM $MgCl_2$, 1.2 mM $CaCl_2$, 10 mM dextrose and 10 mM HEPES (pH = 7.4). To promote circulation, air was bubbled into the buffers, which were maintained at 37 °C. After a stable baseline I_{sc} was achieved, 10 µM forskolin (Selleckchem #S2449) was added to the basolateral chamber to stimulate the generation of cAMP and activate CFTR. Once the current was stabilized at the maximal forskolin-stimulated, 10 µM $CFTR_{inh}$-172 (Selleckchem #S7139) was added to the apical chamber to inhibit CFTR-mediated current. Employing the software Acquire and Analyze (Physiologic Instruments), the data were acquired to compute the CFTR specific function. The contribution of the CFTR specific response to the overall current was calculated in the cells as a change in I_{sc} (ΔI_{sc}) defined as the difference between the maximal stable current stimulated by forskolin and the baseline achieved after adding $CFTR_{inh}$-172. To assess functional CFTR consequences, ΔI_{sc} was normalized based on mRNA quantity to quantify percent-WT function as previously described (7).

Primary nasal cells

Buffer composition was as described previously (9). Apical and basolateral chambers contained the same bathing solution (mM): 115 NaCl, 25 $NaHCO_3$, 5 KCl, 2.5 Na_2HPO_4, 1.8 $CaCl_2$, 1 $MgSO_4$, and 10 dextrose (pH = 7.4). The bath was maintained at 37 °C, and continuously circulated by carbogen gas lift (95% O_2/5% CO_2). After the baseline I_{sc} stabilized, the following inhibitors and activators of I_{sc} were sequentially and cumulatively added: amiloride (100 µM) to inhibit the epithelial Na^+ channel (ENaC); forskolin (10 µM) and 3-isobutyl-1-methylxanthine (IBMX 100 µM) to activate the transepithelial cAMP-dependent current (including Cl^- transport through CFTR channels); and CFTR inhibitor $CFTR_{inh}$-172 (10 µM) to inhibit CFTR. Data were acquired with the software Acquire and Analyze version 2.3.159 (Physiologic Instruments). CFTR specific function in the cells was calculated as change in I_{sc} (ΔI_{sc}) defined as the difference between the sustained phase of the current response after stimulation with forskolin and the baseline achieved after adding $CFTR_{inh}$-172.

Normalization of CFBE cell line function by I_{sc} measurement to WT percentage

Cells were plated for RNA extraction in 6 well plates and cultured for 6 days. The RNA was extracted on the same day as I_{sc} measurement using Nucleospin RNA extraction kit (Macherey-Nagel #740955). Slight modification to kit instructions included lysate collection using TRIZOL (ThermoFisher Scientific #15596026), passing lysate through Qiashdredder column (Qiagen #79656), and nucleic acid purification using chloroform (Fisher Chemical #C298-4) before loading aqueous layer onto the RNA binding columns. 500 ng of total RNA was reverse transcribed using iScript cDNA Synthesis Kit (BioRad #170-8891) for use in quantitative real time PCR. CFTR mRNA expression was measured by qRT-PCR (BioRad - CFX Connect Real-Time System) using SYBR Green Supermix (Biorad) and two primer sets spanning CFTR exon junctions 18-19 and 24-25, primer sequences are shown below:

CFTR qRT primer set 1, e18-e19, 121 bp product

Forward primer 5'- TGACCTTCTGCCTCTTACCA-3'

Reverse primer 5' - CACTATCACTGGCACTGTTGC-3'

CFTR qRT primer set 2, e24-e25, 102 bp product

Forward primer 5'-TCCCTATGAACAGTGGAGTGA-3'

Reverse primer 5'-AGGACAAAGTCAAGCTTCCCA-3'

The housekeeping gene, HPRT1, was used as an expression control, and all cDNA samples were run in triplicate to calculate ΔCt. Any cell line with an expression value outside of the linear expression range described previously (8) was not considered.

CFTR expression is determined by PCR-qRT using two *CFTR* gene primer sets and normalized to *HPRT1* expression level in the same clone. The normalized value is then used to calculate the predicted current that would be generated by WT-CFTR at that level of RNA expression using the correlation between *CFTR* RNA expression and CFTR current generated from independent clones of CFBE cells expressing WT-CFTR. The current generated by the clone under study is then divided by the expected WT-CFTR current to generate the predicted %WT-CFTR function. Specifically, the percent of WT chloride conductance for cells expressing F312del-CFTR was calculated using the following equation: F_{var}=100% X ($I_{sc\text{-}var}$/242.61($mRNA_{var}$)). This was derived from 24 independent cell lines expressing WT-CFTR and accounts for the relationship (slope of the line: 242.61) between chloride channel function ($I_{sc\text{-}var}$) and mRNA level of the variant ($mRNA_{var}$)(7).

Single-channel studies

For patch-clamp experiments, we used mouse mammary epithelial (C127) cells stably expressing wild-type human CFTR (gift of C.R. O'Riordan [Sanofi Genzyme]) (10) and CHO cells co-transfected with F312del-CFTR and enhanced green fluorescent protein N1 (eGFP-N1) using the lipofectamine Plus® system (Thermo Fisher Scientific #L3000001). CHO and C127 cells were cultured and used as described previously (11). C127 cells are the cell line of choice for single-channel studies of wild-type human CFTR because they express low levels of heterologously expressed CFTR (12). The single-channel behavior of human CFTR in excised membrane patches from different mammalian cells is equivalent (13-15).

CFTR Cl^- channels were recorded in excised inside-out membrane patches using an Axopatch 200B patch-clamp amplifier and pCLAMP software (version 10.4) both from Molecular Devices (San Jose, CA, USA) as described previously (11). The pipette (extracellular) solution contained (mM): 140 N-methyl-D-glucamine (NMDG), 140 aspartic acid, 5 $CaCl_2$, 2 $MgSO_4$ and 10 N-tris[hydroxymethyl]methyl-2-aminoethanesulfonic acid (TES), adjusted to pH 7.3 with Tris ([Cl^-], 10 mM). The bath (intracellular) solution contained (mM): 140 NMDG, 3 $MgCl_2$, 1 CsEGTA and 10 TES, adjusted to pH 7.3 with HCl ([Cl^-], 147 mM; free [Ca^{2+}], <10^{-8} M) and was maintained at 37 °C.

CFTR Cl^- channels were activated promptly following membrane patch excision and clamping voltage at –50 mV using the catalytic subunit of protein kinase A (PKA [purified from bovine heart] 75 nM; Calbiochem #539576) and ATP (1 mM; Sigma-Aldrich #A2383). To minimize channel rundown, PKA and ATP were added to all intracellular solutions. In this study, membrane patches contained ≤ 5 active channels determined using the maximum number of simultaneous channel openings, observing the precautions described previously (16) to minimize errors when counting channels. The effects of ivacaftor (Selleckchem #S1144) were studied by addition to the intracellular solution in the continuous presence of ATP (1 mM) and PKA (75 nM). Because of the difficulty of washing ivacaftor from the recording chamber (17), test interventions with ivacaftor were not bracketed by control periods. Instead, they were compared with the pre-intervention control period made with the same concentration of ATP and PKA, but without ivacaftor. After recording, filtering and digitizing data (11), single-channel current amplitude (i), open probability (P_o), mean burst duration (MBD) and interburst interval (IBI) were determined as described (11, 16). On completion of experiments, the recording chamber was thoroughly cleaned before re-use (17).

Statistical analysis

Statistical analysis was performed, and graphs were generated using GraphPad Prism6 (GraphPad Software Inc.). Results are presented as means±SEM, with the number of

experiments indicated. *P* values ≤0.05 were considered significant. Individual-level data underlying each graph and exact *P* values are provided in the supplemental file **Source Data**.

SUPPLEMENTAL MATERIAL: FIGURE and TABLE

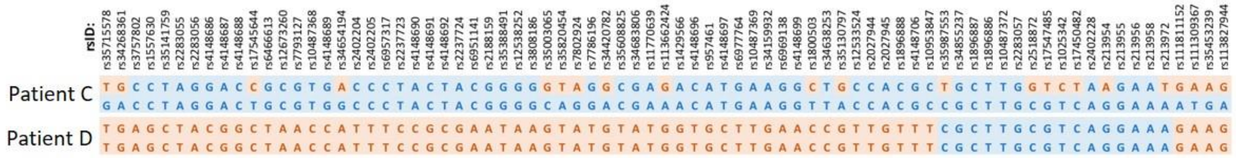

Supplemental Figure 1. Differing *CFTR* haplotypes in two individuals bearing F312del. Patient C is compound heterozygous for F312del and F508del; Patient D is homozygous for F312del and 5T;TG11. Single nucleotide polymorphisms (SNP) identified with by their reference SNP cluster id (rsID) with minor allele frequency > 15% within *CFTR* are shown.

Supplemental Table 1. California Genetic Disease Screening Program CF newborn screening results[A]

Individual	Variant 1 PolyT	Variant 2 PolyT	Age at last contact (y)	Age at diagnosis (y)	Initial sweat chloride (mEq/L)	Highest sweat chloride (mEq/L)	Pancreatic status[B]	Immunoreactive trypsinogen (ng/ml)
1	F312del 9T	F508del 9T	5	<1	75	75	sufficient	80
2	F312del	F508del	2	<1	48	106	sufficient	34[C]
3	F312del	Y1092X	5	<1	112	112	sufficient	91
4	F312del	F508del	5	<1	112	112	sufficient	86
5	F312del	F508del	4	<1	98	98	sufficient	223
6	F312del	406-1G->A	3	<1	79	79	insufficient	111

[A]Children with CF and clinical parameters collected up to 5 years of age or until families were lost to follow-up

[B]Pancreatic status defined by multiple physician reports of sufficiency or prescriptions of pancreatic enzymes and mention of a positive fecal elastase test <200 µg/g

[C]This individual was missed at birth due to an IRT value in the normal range and was reported as having CF to the Genetic Disease Screening Program soon after birth. Redraw resulted in a second IRT with normal result, but sweat chloride was elevated on both initial and subsequent measures.

SUPPLEMENTAL REFERENCES

1. Strom CM, et al. Cystic fibrosis testing 8 years on: lessons learned from carrier screening and sequencing analysis. *Genet Med.* 2011;13(2):166-72.
2. Liu X, et al. ROCK inhibitor and feeder cells induce the conditional reprogramming of epithelial cells. *Am J Pathol.* 2012;180(2):599-607.
3. Liu X, et al. Conditional reprogramming and long-term expansion of normal and tumor cells from human biospecimens. *Nat Protoc.* 2017;12(2):439-51.
4. Gentzsch M, Bet al. Pharmacological Rescue of Conditionally Reprogrammed Cystic Fibrosis Bronchial Epithelial Cells. *Am J Respir Cell Mol Biol.* 2017;56(5):568-74.
5. Cui L, et al. Domain interdependence in the biosynthetic assembly of CFTR. *J Mol Biol.* 2007;365(4):981-94.
6. Gottschalk LB, et al. Creation and characterization of an airway epithelial cell line for stable expression of CFTR variants. *J Cyst Fibros.* 2016;15(3):285-94.
7. Raraigh KS, et al. Functional Assays Are Essential for Interpretation of Missense Variants Associated with Variable Expressivity. *Am J Hum Genet.* 2018;102(6):1062-77.
8. Han ST, et al. Residual function of cystic fibrosis mutants predicts response to small molecule CFTR modulators. *JCI Insight.* 2018;3(14):e121159.
9. Veizis EI, et al. Decreased amiloride-sensitive Na+ absorption in collecting duct principal cells isolated from BPK ARPKD mice. *Am J Physiol Renal Physiol.* 2004;286(2):F244-54.
10. Marshall J, et al. Stoichiometry of recombinant cystic fibrosis transmembrane conductance regulator in epithelial cells and its functional reconstitution into cells in vitro. *J Biol Chem.* 1994;269(4):2987-95.
11. Cai Z, et al. Impact of the F508del mutation on ovine CFTR, a Cl- channel with enhanced conductance and ATP-dependent gating. *J Physiol.* 2015;593(11):2427-46.
12. Sheppard DN, Robinson KA. Mechanism of glibenclamide inhibition of cystic fibrosis transmembrane conductance regulator Cl- channels expressed in a murine cell line. *J Physiol.* 1997;503(Pt 2):333-46.
13. Lansdell KA, et al. Comparison of the gating behaviour of human and murine cystic fibrosis transmembrane conductance regulator Cl- channels expressed in mammalian cells. *J Physiol.* 1998;508(Pt 2):379-92.
14. Chen JH, et al. Direct sensing of intracellular pH by the cystic fibrosis transmembrane conductance regulator (CFTR) Cl- channel. *J Biol Chem.* 2009;284(51):35495-506.

15. Bose SJ, et al. Differential thermostability and response to cystic fibrosis transmembrane conductance regulator potentiators of human and mouse F508del-CFTR. *Am J Physiol Lung Cell Mol Physiol.* 2019;317(1):L71-L86.

16. Cai Z, et al. Differential sensitivity of the cystic fibrosis (CF)-associated mutants G551D and G1349D to potentiators of the cystic fibrosis transmembrane conductance regulator (CFTR) Cl- channel. *J Biol Chem.* 2006;281(4):1970-7.

17. Wang Y, et al. CFTR potentiators partially restore channel function to A561E-CFTR, a cystic fibrosis mutant with a similar mechanism of dysfunction as F508del-CFTR. *Br J Pharmacol.* 2014;171(19):4490-503.
